# High-Throughput Sequencing of mGluR Signaling Pathway Genes Reveals Enrichment of Rare Variants in Autism

**DOI:** 10.1371/journal.pone.0035003

**Published:** 2012-04-27

**Authors:** Raymond J. Kelleher III, Ute Geigenmüller, Hayk Hovhannisyan, Edwin Trautman, Robert Pinard, Barbara Rathmell, Randall Carpenter, David Margulies

**Affiliations:** 1 Center for Human Genetic Research, Massachusetts General Hospital, Boston, Massachusetts, United States of America; 2 Department of Neurology, Massachusetts General Hospital and Harvard Medical School, Boston, Massachusetts, United States of America; 3 Program in Neuroscience, Harvard Medical School, Boston, Massachusetts, United States of America; 4 Correlagen Diagnostics, Inc., Waltham, Massachusetts, United States of America; 5 Seaside Therapeutics, Cambridge, Massachusetts, United States of America; University of Jaén, Spain

## Abstract

Identification of common molecular pathways affected by genetic variation in autism is important for understanding disease pathogenesis and devising effective therapies. Here, we test the hypothesis that rare genetic variation in the metabotropic glutamate-receptor (mGluR) signaling pathway contributes to autism susceptibility. Single-nucleotide variants in genes encoding components of the mGluR signaling pathway were identified by high-throughput multiplex sequencing of pooled samples from 290 non-syndromic autism cases and 300 ethnically matched controls on two independent next-generation platforms. This analysis revealed significant enrichment of rare functional variants in the mGluR pathway in autism cases. Higher burdens of rare, potentially deleterious variants were identified in autism cases for three pathway genes previously implicated in syndromic autism spectrum disorder, *TSC1, TSC2,* and *SHANK3*, suggesting that genetic variation in these genes also contributes to risk for non-syndromic autism. In addition, our analysis identified *HOMER1,* which encodes a postsynaptic density-localized scaffolding protein that interacts with Shank3 to regulate mGluR activity, as a novel autism-risk gene. Rare, potentially deleterious *HOMER1* variants identified uniquely in the autism population affected functionally important protein regions or regulatory sequences and co-segregated closely with autism among children of affected families. We also identified rare ASD-associated coding variants predicted to have damaging effects on components of the Ras/MAPK cascade. Collectively, these findings suggest that altered signaling downstream of mGluRs contributes to the pathogenesis of non-syndromic autism.

## Introduction

Autism is a brain disorder of early childhood characterized by impaired communication, impaired social interaction and restricted and repetitive patterns of activities and interests. The phenotypic breadth of autism is encompassed by the term autism spectrum disorder (ASD), which collectively affects nearly 1% of children, typically with onset prior to the age of three years. Insights into the genetic landscape of ASDs have indicated substantial heterogeneity. Identification of the genes responsible for recognized neurogenetic syndromes with high prevalence of ASD, such as *FMR1* and *TSC1/2*, and localization of causative genes within microdeletions associated with non-syndromic (idiopathic) autism, such as *SHANK3*, *NLGN3,* and *NLGN4*, have highlighted the role of single-gene mutations in disease pathogenesis (reviewed in [1]–[4]). A series of recent genome-wide studies have revealed a significant contribution of rare *de novo* copy number variants (CNVs) affecting many different loci to ASD susceptibility [5]–[9]. These known genetic factors are estimated to account for less than 20% of ASD cases, however, and thus much of the genetic basis of ASDs remains unexplained.

**Figure 1:**
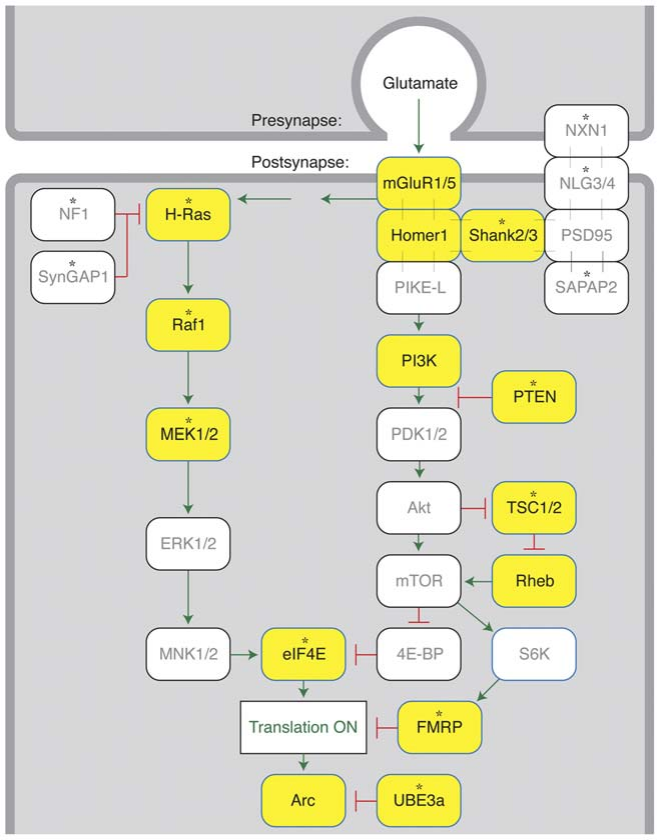
The mGluR pathway coupling synaptic activity to synaptic protein synthesis. The diagram illustrates components and interactions in the mGluR pathway [4]. Genes encoding proteins highlighted in yellow were sequenced in this study. Activation of postsynaptic group 1 mGluRs (mGluR1, mGluR5) stimulates protein synthesis by signaling through the Ras/ERK and PI3K/mTOR pathways. Group 1 mGluR function is modulated by interaction with Homer1, which interacts in turn with Shank3 and links mGluRs to the network of postsynaptic density-localized proteins. FMRP regulates synaptic protein synthesis by binding to target mRNAs and repressing their translation. Arc regulates mGluR-dependent synaptic plasticity, and its levels are regulated by FMRP-dependent translation and Ube3a-dependent degradation. The activity of the mGluR pathway is regulated by several pathway components responsible for syndromic ASDs (indicated by asterisks), including NF1 (neurofibromatosis type 1), Ras/ERK cascade members (cardiofaciocutaneous/Noonan syndromes), PTEN (ASD with microcephaly), TSC1 and TSC2 (tuberous sclerosis complex), FMRP (fragile X mental retardation syndrome), and Ube3a (Angelman's syndrome). Mutations in Shank3, Nrxn1, Nlgn3, and Nlgn4 cause rare non-syndromic ASDs, and structural variants in SynGAP1 and DLGAP2/SAPAP2 have been associated with autism (indicated by asterisks) [7], [23], [43].

Identification of common molecular pathways affected by genetic variation in autism is essential to understand disease pathophysiology and devise effective therapeutic strategies [10]. Increasing evidence suggests a central role for defects in synaptic structure and function in the pathogenesis of autism despite the underlying genetic heterogeneity [3], [11]–[14]. However, the neuronal pathways subserving synapse structure and function which are pathologically altered and which may represent convergence points for genetic lesions in autism remain to be defined. In this study, we tested the hypothesis that functional genetic variation in components of the signaling network coupling group 1 mGluRs to synaptic protein synthesis contributes to the pathogenesis of non-syndromic autism. Several lines of evidence suggest that the “mGluR pathway” may play an important role in ASD pathophysiology [4]. Group 1 mGluRs (mGluR1/5) signal through the Ras/ERK and PI3K/mTOR signaling cascades to regulate protein synthesis (Fig. 1). Syndromic disorders with high prevalence of ASD are caused by mutations in pathway components that regulate ERK activity (*NF1*, *RAS* isoforms, *RAF* isoforms, *MAP2K1*, *MAP2K2*), mTOR activity (*TSC1, TSC2*, *PTEN*) or mRNA translation directly (*FMR1*) [4]. Analysis of *Fmr1^−/Y^* mice, a model of fragile X syndrome, the most common inherited cause of autism, has revealed excessive mGluR5-dependent synaptic protein synthesis and plasticity [15]. An array of phenotypes in these mice could be corrected by attenuation of mGluR5 activity [16]. Similarly, analysis of *Nf1* ^−/−^ and *Tsc2* ^+/−^ mice has suggested treatments to correct behavioral impairment in neurofibromatosis and tuberous sclerosis complex (TSC) [17], [18]. Thus, understanding how genetic variation in the mGluR pathway contributes to non-syndromic autism may suggest new therapeutic interventions since this pathway is amenable to pharmacological manipulation.

**Figure 2:**
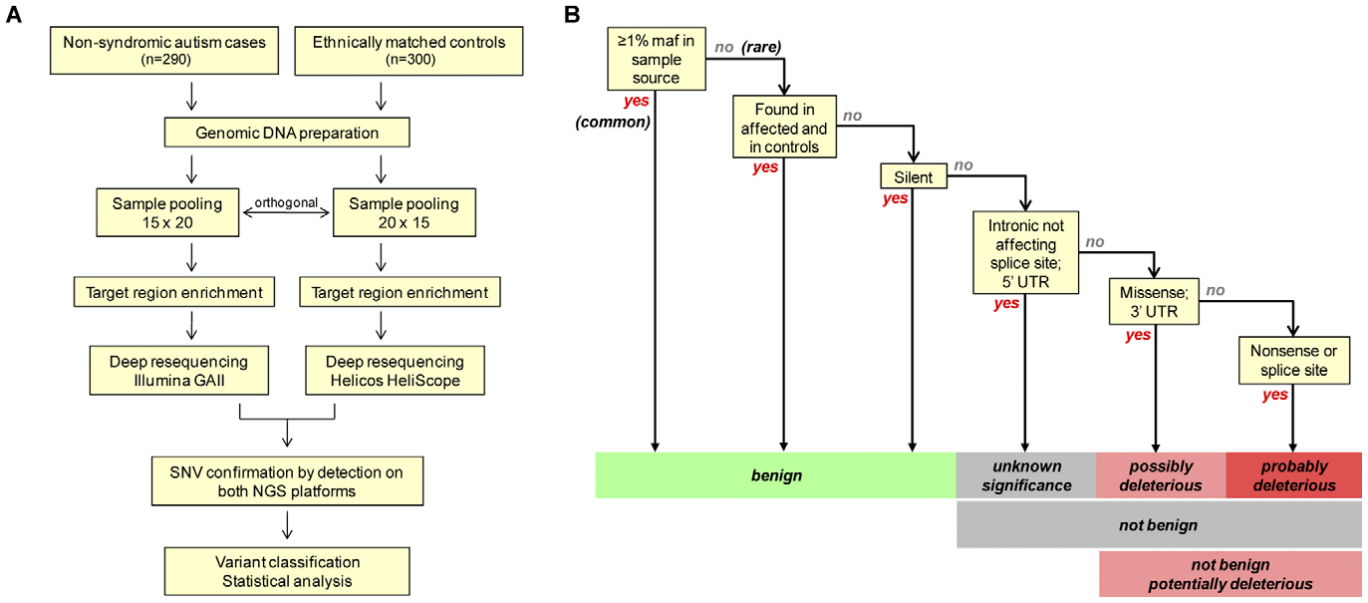
SNV detection and classification. (A) The flow diagram depicts the experimental strategy for SNV discovery and confirmation. For the AGRE and control cohorts, orthogonal multiplexing was performed to prepare two distinct sets of sample pools (15 pools of 20 samples each, or 20 pools of 15 samples each). Following enrichment of exonic target regions for all 18 mGluR pathway genes, SNVs were identified and confirmed by deep resequencing of orthogonal pools on two independent NGS platforms (Illumina GAII and the Helicos HeliScope). SNVs concordantly detected on both platforms were then analyzed as shown in panel B. (B) The flow diagram depicts the procedure used to classify the presumptive functional effects of identified variants. SNVs concordantly detected on both NGS platforms were classified as common or rare using a minor allele frequency (maf) threshold of 1%. Common SNVs, rare SNVs occurring in both autism and control populations, and rare synonymous (silent) SNVs were considered likely to be benign. Rare SNVs in intronic sequences flanking exons that did not affect conserved splice donor or acceptor sites or in 5′ untranslated regions were classified as not benign but of unknown significance. Rare SNVs causing missense substitutions or occurring in mRNA 3′ untranslated regions (and therefore possibly affecting mRNA stability or translation [28], [29]) were considered possibly deleterious, and rare SNVs causing nonsense mutations or affecting conserved splice donor or acceptor sequences were considered probably deleterious. These latter two categories of SNVs were together considered potentially deleterious.

To test the hypothesis that genetic variation in the mGluR pathway increases risk for non-syndromic autism, we took advantage of the massively parallel sequencing capacity offered by next-generation sequencing (NGS) technologies to interrogate a panel of candidate genes in a cohort of autism cases and controls. Our strategy was designed to enhance high-throughput discovery by performing variant discovery in pools of samples (Fig. 2A). We then resequenced “orthogonal” pools on a second next-generation platform to validate variants and assign them to individual samples. We applied this approach to identify variants associated with autism in coding exons or flanking sequences in a panel of 18 genes encoding key components of the mGluR pathway [4] (Fig. 1; Table S1). Genes selected for analysis encode group 1 mGluRs (mGluR1, mGluR5), postsynaptic density (PSD)-associated scaffolding proteins that regulate mGluR function (Homer1, Shank3), components of the Ras/ERK (H-Ras, Raf, MEK1, MEK2) and PI3K/mTOR (PI3K catalytic and regulatory subunits, PTEN, TSC1, TSC2, Rheb) signaling cascades, mRNA-binding factors that regulate protein synthesis (eIF4E, FMRP), a protein target of mGluR-induced translation that regulates synaptic plasticity (Arc), and an E3 ubiquitin ligase known to regulate Arc degradation (Ube3a) [19]. Although some of the genes studied are known to cause syndromic ASD (*e.g. FMR1, TSC1, TSC2, UBE3A*), their possible role in non-syndromic autism has been unclear.

## Results

We analyzed single-nucleotide variants (SNVs) in mGluR pathway genes by high-throughput sequencing in a cohort of 290 unrelated AGRE cases and 300 ethnically-matched controls (Fig. 2A; Table S2). Inclusion criteria for AGRE cases were a diagnosis of idiopathic (“non-syndromic”) autism and at least one affected sibling. AGRE cases are screened to exclude non-idiopathic (“syndromic”) autism secondary to known neurogenetic disorders such as fragile-X syndrome [20]. Two distinct sets of pools were prepared from genomic DNA samples isolated from these cohorts for sequencing on the Illumina GAII and the Helicos HeliScope. An orthogonal strategy for sample pooling was used in which samples were arrayed in a matrix with 15 rows and 20 columns. Samples were pooled along rows to generate 15 pools of 20 samples each for GAII sequencing and pooled along columns to generate 20 pools of 15 samples each for Heliscope sequencing. Each sample representing a single case or control subject was thus identified by a unique combination of two pools representing its unique position within this matrix. Each genomic DNA pool was used as a template for PCR amplification of all coding exons from our panel of 18 mGluR pathway genes (240 exons comprising a total of 40,473 bases). PCR amplicons from each genomic DNA pool were concatenated and sheared to construct libraries for high-throughput sequencing. The average coverage per exon per pool was 610 for the GAII and 1,688 for the HeliScope. An average coverage of ≥10 per individual was achieved for 87% (GAII) and 97% (HeliScope) of exons across all pools. Sensitivity of variant detection was therefore generally limited by the lower coverage achieved on the GAII in this analysis.

We used confirmation by Sanger sequencing to evaluate the sensitivity and fidelity of SNV detection on each NGS platform and on both platforms combined. The false discovery rate for single variant occurrences (singletons) fell sharply without losing sensitivity when considering only SNVs detected concordantly on both platforms (Fig. S1). We therefore limited our subsequent analysis to SNVs that were concordantly detected on both platforms.

Common SNVs were defined as those with allele frequencies ≥1%, while rare SNVs were defined as having allele frequencies <1%. The number of common SNVs did not differ between the AGRE and control groups (Table 1). Rare SNVs were modestly enriched in AGRE samples compared to controls (302 and 276, respectively). However, when we excluded rare SNVs that are either silent or present in both populations, and therefore presumed to be benign (Fig. 2B), a significant enrichment of genetic variation was detected in the autism population, with 80 and 49 SNVs in AGRE and control groups, respectively *(P = *0.001). When we additionally eliminated SNVs located in 5′ UTRs or deep intronic regions, thereby focusing on SNVs with potentially deleterious effects, there emerged a two-fold enrichment of variants in the autism population, with 58 and 29 SNVs in the AGRE and control groups, respectively (*P* = 0.0005), occurring in 49 and 32 individuals, respectively (Table 1, Table 2, Table S3). The two-fold enrichment of SNVs in the autism population persisted if we further excluded SNVs characterized as common in dbSNP (build 132), with 57 and 27 SNVs in the AGRE and control populations, respectively (*P* = 0.0002).

**Table 1:** Enrichment of rare functional variants in mGluR pathway genes in autism cases detected by high-throughput sequencing.

Variant	Common		Rare		Rare		Rare,	
Type:					Not benign		Not benign	
							Pot. deleterious	
Gene	AGRE	Ctrl	AGRE	Ctrl	AGRE	Ctrl	AGRE	Ctrl
*ARC*	2	2	6	9	3	1	3	1
*EIF4E*	1	1	3	4	1	0	0	0
*FMR1*	2	3	3	7	0	3	0	2
*GRM1*	7	7	20	25	2	7	2	6
*GRM5*	22	21	31	29	7	7	3	6
*HOMER1*	4	4	8	2	7	0	**5**	**0**
*HRAS*	3	3	3	2	1	0	1	0
*MAP2K1*	2	3	5	4	0	1	0	1
*MAP2K2*	5	6	21	19	4	1	3	1
*PIK3CA*	16	16	8	4	2	2	1	2
*PIK3R1*	6	6	8	7	4	3	1	0
*PTEN*	1	1	4	4	0	0	0	0
*RAF1*	1	0	7	8	2	2	2	2
*RHEB*	1	1	1	1	0	0	0	0
*SHANK3*	7	6	80	60	19	4	**14**	**3**
*TSC1*	3	5	26	13	11	1	**8**	**0**
*TSC2*	14	12	61	70	16	15	**14**	**5**
*UBE3A*	2	1	7	8	1	2	1	0
All genes	99	98	302	276	80	49	**58**	**29**

SNVs in mGluR pathway genes were identified in pools of AGRE or control samples and classified based on allele frequency and predicted functional impact. The values shown represent the numbers of distinct variants identified. A significant excess of rare, potentially deleterious variants in the AGRE group relative to the control group was observed for the *HOMER1*, *SHANK3*, *TSC1*, and *TSC2* genes (highlighted in bold).

**Table 2:** Rare, potentially deleterious variants identified in mGluR pathway genes in autism cases.

*ARC*	*GRM1*	*GRM5*	*HOMER1*	*HRAS*	*MAP2K2*	*PIK3CA*	*PIK3R1*	*RAF1*	*SHANK3*	*TSC1*	*TSC2*	*UBE3A*
c.587C>G	c.1882C>G	c.727G>T	c.195G>T	c.383G>A	c.581-1G>T	c.1544A>G	c.889G>A	c.122G>A	c.612C>A	c.346T>G	c.433G>A	c.333C>G
(P196R)	(R628G)	(A243S)	(M65I)	(R128Q)	(splice donor)	(N515S)	(E297K)	(R41Q)	(D204E)	(L116V)	(A145T)	(N111K)
c.605A>T	c.2051G>A	c.1417G>C	c.290C>T		c.889C>T			c.356C>T	c.763C>T	c.692C>T	c.1292C>T	
(D202V)	(R684H)	(E473Q)	(S97L)		(R297W)			(A119V)	(H255Y)	(P231L)	(A431V)	
c.829G>A		c.3503T>C	c.425C>T		c.1217C>T				c.898C>T	c.1006C>T	c.1912G>A	
(G277S)		(L1168P)	(P142L)		(3′ UTR)				(R300C)	(R336W)	(V638M)	
			c.968G>A						c.920C>G	c.1178C>T	c.2155T>C	
			(R323H)						(A307G)	(T393I)	(Y719H)	
			c.1080C>T						c.1315C>T	c.1342C>T	c.2621C>T	
			(3′ UTR)						(P439S)	(P448S)	(P874L)	
									c.1337G>T	c.1580A>G	c.3252C>G	
									(G446V)	(Q527R)	(D1084E)	
									c.3761C>T	c.1960C>G	c.3827C>T	
									(A1254V)	(Q654E)	(S1276F)	
									c.3764C>T	c.2718A>C	c.3914C>T	
									(P1255L)	(Q906H)	(P1305L)	
									c.3836C>T		c.3974G>A	
									(P1279L)		(G1325D)	
									c.4025C>T		c.4051G>A	
									(P1342L)		(E1351K)	
									c.4405G>C		c.4316G>A	
									(G1469R)		(G1439D)	
									c.4406G>T		c.4460C>G	
									(G1469V)		(S1487C)	
									c.4490G>A		c.5429G>A	
									(R1497Q)		(3' UTR)	
									c.4720G>A		c.5450G>A	
									(G1574R)		(3′ UTR)	

Table 2 summarizes the 58 rare, potentially deleterious SNVs that were identified in mGluR pathway genes only in autism cases. For each variant, the nucleotide substitution is shown, and the corresponding amino acid substitution is indicated parenthetically.

We applied several commonly used computational tools for pathogenicity prediction to assess the functional impact of the rare, potentially deleterious missense variants identified in the AGRE and control groups. Since the performance and reliability of methods for pathogenicity prediction varies widely, and their results typically correlate poorly [21], we compared the predictions derived from SIFT, PolyPhen2, SNP&GO and MutPred. Although SIFT did not predict a significant difference between groups, PolyPhen2, SNP&GO, and MutPred each predicted a 2- to 3-fold enrichment of damaging missense variants in the AGRE group. Overall, comparably high proportions of the variants classified as potentially deleterious in the AGRE and control groups (68% and 71%, respectively) were predicted to be functionally damaging by at least one of the four prediction tools, supporting the notion that disruptive variants within mGluR pathway components occur at a higher rate in the autism relative to the control population.

At the level of individual pathway genes, we identified a significant excess of SNVs in the autism population for the *TSC1*, *TSC2*, *SHANK3,* and *HOMER1* genes (*P*<0.05). Causal roles for *TSC1* and *TSC2* have previously been demonstrated in syndromic autism. *TSC1* or *TSC2* mutations can cause TSC, a syndromic disorder characterized by tumor growth in multiple organs, including the brain. Although the manifestations of TSC include ASD in up to 50% of cases [22], our findings additionally implicate *TSC1* and *TSC2* as risk genes for non-syndromic autism independent of their causative role in TSC. Consistent with this view, the majority of the rare, potentially disruptive *TSC1/TSC2* SNVs identified in the AGRE population are novel, and none of these SNVs has been identified previously as a cause of TSC (http://chromium.liacs.nl/LOVD2/TSC/home.php). Our identification of increased genetic variation in *SHANK3* in autism cases supports the emerging view of *SHANK3* as an important autism-risk gene [23]. One missense variant observed in our study (R300C) was previously identified as a potential risk factor for ASD [23]. In addition, we identified a number of novel rare *SHANK3* SNVs in the autism population (Table 2). The over-representation of rare, potentially disruptive variants in genes previously implicated in ASD (*TSC1*, *TSC2*, *SHANK3*) provides validation of this approach to detect genes that contribute genetic risk in autism.

**Figure 3:**
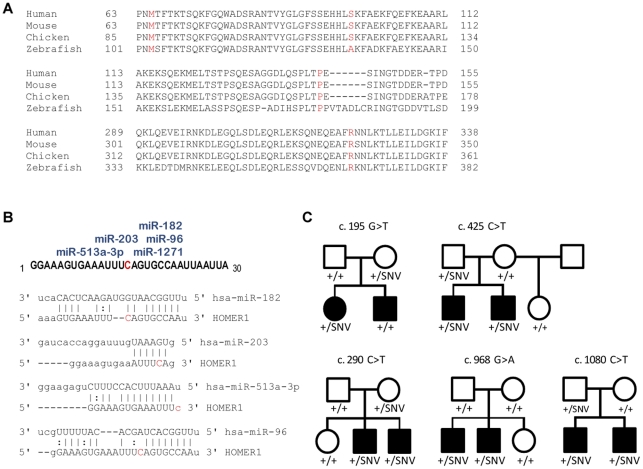
Autism-specific *HOMER1* variants affect conserved residues or microRNA binding sites and co-segregate with autism. (A) Multiple sequence alignments are shown for three segments of the Homer1 protein that contain missense substitutions caused by autism-specific SNVs identified in this study. The amino acid residues altered by these substitutions (highlighted in red) are highly conserved across mammalian and/or vertebrate evolution. (B) The autism-specific *HOMER1* c.1080C>T variant is predicted to alter multiple microRNA-binding sites in the *HOMER1* 3′ UTR. The sequence of the *HOMER1* 3′ UTR is shown at top (the c.1080 position 15 nucleotides distal to the translation stop codon highlighted in red), together with a cluster of microRNA binding sites predicted by the miRanda and Microcosm applications (miRanda target prediction based on ≥ 6-mer seed complementarity and mirSVR score ≤0.1) [32], [33]. Predicted pairing between specific microRNAs and the *HOMER1* 3′ UTR that would be altered by the c. 1080 C>T variant is shown at bottom. (C) Co-segregation with autism was analyzed for the rare, potentially deleterious *HOMER1* missense variants uniquely identified in AGRE probands by genotyping available parents and siblings. Filled symbols indicate a diagnosis of autism or ASD; unfilled symbols indicate reportedly unaffected individuals. Genotypes are shown for each individual, with “+” designating the wild-type allele and “SNV” designating the indicated variant allele.

The fourth gene displaying a significant enrichment of autism-associated rare SNVs, *HOMER1*, has not previously been implicated in autism. Homer1 is a PSD-localized scaffolding protein that interacts with a variety of PSD proteins, including mGluRs and Shank proteins [24]. Binding of Homer1 to mGluRs promotes trafficking of mGluRs to the postsynaptic membrane and couples mGluR5 to the mTOR signaling pathway [25]. Homer and Shank proteins interact to form an extended polymeric platform required for recruitment and assembly of synaptic proteins and structural integrity of dendritic spines [26]. Consistent with this function, the Homer-Shank interaction has been shown to promote morphological and functional maturation of dendritic spines [27]. We identified multiple rare missense variants in *HOMER1* in AGRE cases but not in controls. All of the identified missense variants in *HOMER1* alter residues that are invariant among mammalian species, and all but one is invariant across vertebrate species (Fig. 3A). Two of these variants (c.195G>T, M65I and c.290C>T, S97L) localize to the EVH1 (Ena/VASP homology 1) domain of Homer1, which binds to Pro-Pro-Ser-Pro-Phe motifs in mGluR1 and mGluR5 and a Pro-Pro-Glu-Glu-Phe motif in Shank3 [24]. A third potentially damaging SNV in *HOMER1* (c.425C>T, P142L) affects one of the conserved prolines within the P-motif of the CRH1 (conserved region of Homer 1) domain, which serves as an internal binding site for the EVH1 domain. It has been proposed that the P-motif competes for binding of the Homer1 EVH1 domain to the proline-rich motif in target proteins such as mGluRs, thereby modulating Homer1 homo-multimerization and mGluR interaction. Interestingly, one of the *GRM5* variants (c.3503T>C, L1168P) detected in AGRE samples is located in close proximity to the conserved Pro-Pro-Ser-Pro-Phe Homer1-binding motif in mGluR5. In addition, we identified an SNV in the *HOMER1* 3′ UTR (c. 1080 C>T) only in the autism and not in the control population. Growing evidence suggests an important role for 3′ untranslated regions (UTRs) as the sites of pathogenic variation due to their diversity and density of *cis*-acting regulatory elements [28], [29]. In particular, genetic variants that alter microRNA-binding sites have been implicated in the pathogenesis of a variety of human diseases, including the neuropsychiatric disorder Tourette's syndrome [30], [31]. The identified *HOMER1* 3′ UTR variant, which is located 15 nucleotides distal to the translation termination codon, lies within a cluster of predicted microRNA binding sites and alters predicted seed pairing for several microRNAs, including miR-96, miR-182, miR-203, and miR-513a-3p (miRanda and Microcosm algorithms, www.microrna.org; www.ebi.ac.uk/enright-srv/microcosm [32], [33]) (Fig. 3B). Based on the predicted effects on microRNA binding, this variant may perturb the efficiency and/or tissue specificity of *HOMER1* mRNA translation and protein expression.

To assess further the pathogenicity of the rare, potentially disruptive *HOMER1* variants uniquely identified in the autism population, we analyzed co-segregation of these variants with autism (Figure 3C). Parents and siblings of probands in the families carrying each of the five *HOMER1* variants were genotyped for the relevant *HOMER1* variant as well as any other rare variants detected in the proband. Four of the variants (c. 290C>T, c.425C>T, c. 968G>A, and c.1080 C>T) co-segregated perfectly with the autism phenotype in affected and unaffected children. Probands from two of these families carried a second rare variant in addition to the *HOMER1* variant, but these other variants did not co-segregate with the autism phenotype (*HOMER1* c.290C>T and *SHANK3* c.898C>T; *HOMER1* c.195 and *PIK3CA* c.2294+19C>T). The fifth *HOMER1* variant (c.195G>T, the only *HOMER1* variant carried by a female proband) was not detected in an affected sibling, suggesting that this variant may modify autism risk. Interestingly, the c.968G>A variant was present in two affected male children but absent in both parents, suggesting that this variant arose *de novo* in one of the parental germlines. This finding is consistent with increasing evidence that *de novo* CNVs and SNVs with high penetrance play major roles in autism [6], [34], [35]. The remaining four variants were transmitted to affected children by unaffected carriers, possibly reflecting incomplete penetrance of pathogenic variants among parents in families with multiplex autism [34], [36].

Although significant enrichment of rare, potentially disruptive variants in AGRE samples relative to controls was limited to the *TSC1, TSC2, SHANK3,* and *HOMER1* genes, individual variants in additional genes suggest a role for the Ras/ERK cascade in autism susceptibility. One AGRE sample harbored an SNV in *MAP2K2* (c.581-1G>T) that alters a conserved splice-acceptor site; skipping of the adjoining exon would result in a frameshift mutation within the kinase domain and is thus highly likely to be damaging. Familial segregation analysis revealed the presence of this variant in a non-affected as well as an affected sibling, indicating reduced penetrance. A potentially damaging missense variant was also detected in *HRAS* (c.383G>A, R128Q); this substitution alters a highly conserved basic residue required for interaction of GTP-bound H-Ras with the plasma membrane and Raf [37]. Familial segregation analysis revealed absence of this variant in an affected sibling of the proband, suggesting a modifying rather than causal role for this variant. Mutations in *MAP2K2* and *HRAS* are responsible for cardiofaciocutaneous (CFC) and Costello syndrome, respectively, related monogenic disorders characterized by mental retardation, facial dysmorphism, cardiac defects and a high prevalence of autistic features [38], [39]. CFC and Costello syndromes are thought to be caused by gain-of-function mutations that activate the Ras/ERK pathway, whereas the *MAP2K2* and *HRAS* variants that we identified in autism cases are most compatible with loss of protein function. These findings raise the possibility that rare genetic variation within the Ras/ERK cascade may contribute to non-syndromic autism risk independent of this pathway's role in CFC and Costello syndromes.

## Discussion

The findings reported here lend strong support to the hypothesis that perturbed function of the signaling pathways coupling mGluRs to synaptic protein synthesis plays an important role in autism pathogenesis. Overall, we identified rare, potentially damaging variants in 20% (58/290) of the autism cases analyzed but in only 10% (29/300) of controls, suggesting a substantial contribution of rare functional genetic variation within the mGluR pathway to autism susceptibility. A series of recent genome-wide studies has highlighted the association of rare CNVs with autism [5]–[9], [36], [40]. Our findings complement these studies by pointing to an important role for rare SNVs in pathophysiogically relevant genes in autism. Consistent with this view, exome sequencing of 20 trios with idiopathic ASD revealed potentially causative *de novo* SNVs in several genes known to be associated with autism, intellectual disability and epilepsy [35]. Interestingly, our analysis revealed a significant excess of rare, potentially deleterious variants in three known autism genes, *SHANK3*, *TSC1*, and *TSC2*, in individuals with non-syndromic autism. Whereas mutations in the *TSC1* and *TSC2* genes typically cause syndromic autism in the context of TSC, our results suggest that these genes contribute to non-syndromic autism risk independent of their causal role in TSC. Mutations in *SHANK3* have previously been reported as rare monogenic cause of non-syndromic autism and syndromic autism (in the context of the 22q13.3 microdeletion syndrome) [23]. A recent study also identified a number of SNVs in the *TSC1, TSC2*, and *SHANK3* genes in simplex autism cases (although the frequency of SNVs in these genes in controls was not reported) [41]. Taken together, these findings further the view that non-syndromic and syndromic forms of autism share common pathophysiological mechanisms.

The identification of *HOMER1* as an autism risk gene adds an important component to the network of PSD proteins causally implicated in autism, which now includes the products of the *SHANK2* [7], [42], *SHANK3* [23], *NLGN3* [43], *NLGN4* [43], *SYNGAP1* [7], and *DLGAP2* [7] genes (Fig. 1). A pathogenic role for *HOMER1* variation in autism is supported by several observations: multiple rare, potentially deleterious variants were identified in the AGRE population but not in the control population; these autism-specific *HOMER1* missense variants affect functionally significant protein motifs or regulatory sequences; these autism-specific variants also display tight co-segregation with autism in children of affected families, including in one case a *de novo HOMER1* variant present in two affected children. The Homer1 protein interacts with mGluRs and Shank proteins and couples mGluR5 to the mTOR signaling pathway [24], [25]. Homer1 thus provides a novel link between autism-associated gene products that operate downstream of mGluRs and those that interact with Shank proteins, most notably neuroligins [12]. In addition, the Homer-Shank interaction scaffolds and regulates the function of group 1 mGluRs, NMDA receptors and AMPA receptors [24], key glutamate receptor subtypes that mediate synaptic plasticity. Intriguingly, recent evidence indicates that disrupted mGluR5-Homer1 interactions in a mouse model of FXS (*Fmr1^-/Y^* mice) underlies the development of phenotypes relevant to autism, including hippocampal protein synthesis, neocortical circuit dysfunction and behavior [44]. The association of rare functional variation in Homer1 with genetic risk for autism identified in this study provides further insight into the emerging role of synaptic dysfunction in autism pathogenesis [3], [11]–[14].

Our identification of rare damaging variants in the *HRAS* and *MAP2K2* genes further suggests that dysregulation of the Ras/ERK pathway may contribute to autism risk. Consistent with this notion, mutations have been identified in *SYNGAP1* in both ASDs and non-syndromic mental retardation [7], [45]. SynGAP1, which interacts with the PSD scaffold through PSD-95, negatively regulates Ras/ERK pathway activity, synaptic delivery of AMPA receptors and synaptic transmission [46]. Prior studies have shown that ERK activation is required for synaptic activity-induced protein synthesis, mGluR-dependent synaptic plasticity and cognitive function [47]–[49].

Collectively, our findings support the view that genetic susceptibility in autism is attributable to the cumulative contribution of individually rare variants in components of the signaling network that couples PSD proteins and downstream effector mechanisms to synaptic function. Furthermore, our results suggest that pharmacological modulation of the signaling mechanisms coupling mGluRs to synaptic protein synthesis may be an effective therapeutic strategy in autism, and that treatments developed for syndromic forms of autism may apply more broadly to non-syndromic autism.

## Materials and Methods

### Sample selection

Genomic DNA prepared from blood samples was obtained for all unrelated individuals in the AGRE (Autism Genetic Resource Exchange) collection [20] who satisfied the diagnostic criteria of idiopathic (non-syndromic) autism by the Autism Diagnostic Interview, Revised (ADI-R), and at least one sibling with ASD (n = 290 total). Genomic DNA prepared from blood samples was obtained from the Coriell collection for healthy control individuals (n = 300). Relevant demographic and diagnostic features of the AGRE and control cohorts are summarized in Table S2. The sex ratios in the AGRE and control groups approximate the sex ratios in the autism and neurotypical populations (4∶1 and 1∶1, respectively). Females are less likely than males to express an ASD phenotype, and pathogenic variants may display decreased penetrance in female carriers [34], [36]. Since we considered variants detected in both the control and AGRE populations to be non-pathogenic, over-representation of females in the control group relative to the AGRE group could lead to undercalling of variants as pathogenic, but is unlikely to lead to overcalling. We did not observe any significant differences in the rates of detection of rare, potentially deleterious variants between males and females in either the AGRE or control groups (variant rates: AGRE males 19% (44/232), AGRE females 19% (11/58); control males 8.5% (12/141), control females 7.5% (12/159)). These sex-stratified data also demonstrate significantly higher rates of rare, potentially deleterious variants in both AGRE males and AGRE females relative to their control counterparts. The secondary analysis of existing, de-identified samples and data from the AGRE and Coriell repositories conducted in this study was considered exempt from IRB review.

### Next-generation sequencing

DNA concentration was determined for all samples using Nanodrop (Thermo Fisher Scientific). Equal amounts of control sample DNAs were combined into orthogonal pools of 20 samples each and 15 samples each. The same pooling strategy was applied to the AGRE samples; since only 290 AGRE samples were available, 10 samples were included in two different 20-sample and two different 15-sample pools. Each pool then served as the template for PCR amplification of all coding exons comprising the longest isoform expressed by each of the 18 candidate genes, using specific PCR primers tailed at the 5′ end with a 14-bp sequence containing a Not1 restriction site. All PCR products derived from the same template (*i.e.* sample pool) were pooled, digested with Not1, and ligated to form concatemers, which were subsequently randomly sheared into fragments with a mean size of 150 to 300 bp using a Covaris S2 instrument (Covaris, Woburn, MA). These fragments were prepared for sequencing on an Illumina Genome Analyzer II (GAII, Illumina, San Diego, CA) (20-sample pools) or a Helicos HeliScope (Helicos Biosciences, Cambridge, MA) (15-sample pools) according to the manufacturers' instructions. Illumina sequencing was performed for 50 cycles, resulting in a read length of ∼50 bases, and HeliScope sequencing was performed for 120 cycles or 30 quads, resulting in an average read length of 32 bases.

### Analysis pipeline for next-generation sequencing data

Reads were aligned to a reference sequence comprising the hg18-derived sequence of each amplified exon with 30 flanking intronic (non-coding) bases on each side. The aligner MOSAIK was used for the GAII reads (https://code.google.com/p/mosaik-aligner/), and the open source aligner IndexDP for the HeliScope reads (http://open.helicosbio.com). Variant calling was performed with GigaBayes for the GAII reads without invoking the Bayesian-based algorithm (http://bioinformatics.bc.edu/marthlab/GigaBayes), and with SNPsniffer for the HeliScope reads (http://open.helicosbio.com). No minimum minor allele frequency threshold was set in GigaBayes, while in SNPsniffer a minimum allele frequency of 1% was used because variant calls became largely unspecific below that threshold. In both cases, variant calls were only accepted if they occurred at least once on each DNA strand. No other filters were used during the initial variant calling. In the subsequent analysis, GAII variant calls were compared to HeliScope variant calls, and all variants called on both the GAII (at a frequency of at least 0.5%) and the HeliScope (at a frequency of at least 1%) were considered confirmed. Confirmed variants occurring in only one pool on each platform could be assigned to individual samples, based on the orthogonal construction of the GAII and HeliScope sample pools. All rare variants discussed in the text were additionally confirmed by Sanger sequencing.

### Sanger sequencing

Sanger sequencing was performed for selected exons in selected samples to confirm rare variants detected during NGS. PCR primers and conditions were the same as those used for amplification of exons from sample pools, except that individual samples rather than pooled samples served as template. Each PCR product was then cycle-sequenced using BigDye Terminator v3.1 reagents (Applied Biosystems, Foster City, CA), with the specific PCR primers serving as sequencing primers, and the sequencing products were separated on an 3730xl Genetic Analyzer (Applied Biosystems). Sequencing traces were visualized using Sequence Scanner software (Applied Biosystems), and presence or absence of a given mutation determined by manual comparison to the reference sequence.

### Validation of variant confirmation strategy

To determine sensitivity of variant detection and false discovery rate in pools on the individual sequencing platforms, we constructed validation pools from 20 (GAII) and 15 (HeliScope) samples in which Sanger sequencing had previously been performed on all coding exons of the genes *MYBPC3*, *MHY7*, *TNNT2*, and *TNNI3*. Each pool was enriched for these targets by PCR amplification, and libraries were constructed and sequenced as described above. The GAII pool contained 17 singleton substitutions, and the HeliScope pool contained 25 singleton substitutions. To determine sensitivity and false discovery rate for the GAII-HeliScope cross-confirmation strategy, we randomly selected 102 singleton variants detected in only one AGRE or only one control pool on each platform, which could be assigned to individual samples based on the unique combination of GAII and HeliScope pools in which the variant was detected. These samples were then subjected to Sanger sequencing to test for presence of the expected variant.

### Variant analysis

All variants identified on both the GAII and HeliScope platforms were classified according to the scheme shown in Figure 2B. Variants identified on one NGS platform but not on the other were excluded from the current analysis. Fisher's exact test was used to determine the significance of differences in variant accumulation between AGRE and control populations, with nominal statistical significance defined as a two-sided *P*<0.05. Input values for Fisher's exact test were calculated from number of subjects tested and number of distinct potentially deleterious variants identified, under the simplifying assumptions that each rare variant occurred only once and no two variants co-occurred. Comparison with computational predictions of pathogenicity for missense variants was performed using the SIFT, PolyPhen2, MutPred and SNPs&GO programs [50]–[53].

## Supporting Information

Figure S1 **Receiver-operating characteristic curves for sensitivity of variant detection and false discovery rate.** The receiver-operating characteristic (ROC) curves show the sensitivity of detecting singleton variants in 20-sample pools (GAII) and/or 15-sample pools (HeliScope) as a function of the false discovery rate (FDR). Each point represents sensitivity and FDR at a different threshold value for the minimal allowed allele frequency in the pool. For detection on both platforms, the allele frequency threshold was varied only for GAII data and kept constant at 1% for HeliScope data. When allele frequency cut-offs of 0.5% (GAII) and 1% (HeliScope) were applied to detection on both platforms, a sensitivity of 99% was achieved for singleton detection at high coverage with a false discovery rate of 11%, thereby eliminating most false-positive variants.
(TIF)

Table S1 **Components of the mGluR signaling pathway analyzed in this study.** Gene and protein names, cytogenetic localization and protein function are listed for the 18 genes encoding mGluR pathway components that were subjected to next-generation sequencing in a cohort of autism cases and controls in this study.
(DOC)

Table S2 **Cohort demographic and diagnostic characteristics.** The demographic and diagnostic features of the AGRE and control cohorts analyzed in this study are summarized.
(DOCX)

Table S3 **Rare variants detected in AGRE and/or control groups.** All SNVs in 18 mGluR pathway genes that were concordantly detected on both NGS platforms are listed and annotated in terms of their location and consequence, occurrence in AGRE and/or control groups, minor allele frequency (maf), and functional significance as outlined in Fig. 2B.
(PDF)
