# Supplementary figures and images for: High-Throughput Sequencing of mGluR Signaling Pathway Genes Reveals Enrichment of Rare Variants in Autism
Source: PLoS One. 2012 Apr 27;7(4):e35003. doi: 10.1371/journal.pone.0035003 (PMC3338748; doi:10.1371/journal.pone.0035003)

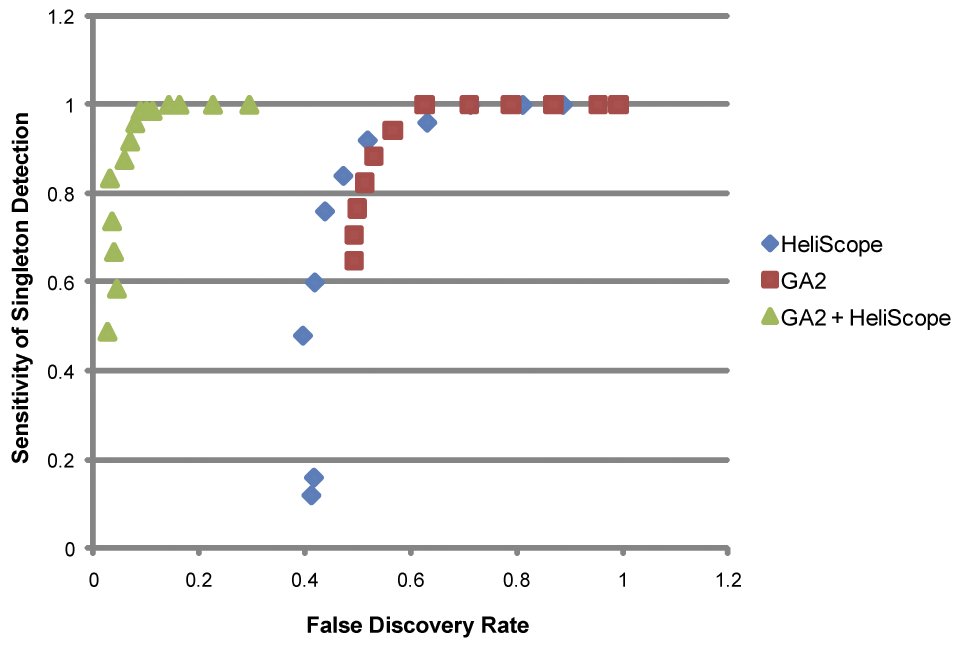

Supplement: Figure S1 — Receiver-operating characteristic curves for sensitivity of variant detection and false discovery rate. The receiver-operating characteristic (ROC) curves show the sensitivity of detecting singleton variants in 20-sample pools (GAII) and/or 15-sample pools (HeliScope) as a function of the false discovery rate (FDR). Each point represents sensitivity and FDR at a different threshold value for the minimal allowed allele frequency in the pool. For detection on both platforms, the allele frequency threshold was varied only for GAII data and kept constant at 1% for HeliScope data. When allele frequency cut-offs of 0.5% (GAII) and 1% (HeliScope) were applied to detection on both platforms, a sensitivity of 99% was achieved for singleton detection at high coverage with a false discovery rate of 11%, thereby eliminating most false-positive variants. (TIF) [file pone.0035003.s001.tif]
