# Supplementary material for: High-Throughput Sequencing of mGluR Signaling Pathway Genes Reveals Enrichment of Rare Variants in Autism
Source: PLoS One. 2012 Apr 27;7(4):e35003. doi: 10.1371/journal.pone.0035003 (PMC3338748; doi:10.1371/journal.pone.0035003)
Supplement: Table S1 — Components of the mGluR signaling pathway analyzed in this study. Gene and protein names, cytogenetic localization and protein function are listed for the 18 genes encoding mGluR pathway components that were subjected to next-generation sequencing in a cohort of autism cases and controls in this study. (DOC) [file pone.0035003.s002.doc]

| **Gene** | **Cytogenetic**  **location** | **Protein** | **Function** |
| --- | --- | --- | --- |
| *ARC* | 8q24.3 | Arc/Arg3.1 | AMPAR endocytosis |
| *EIF4E* | 4q21-25 | eIF4E | translation initiation |
| *FMR1* | Xq27.3 | FMRP | translational repression |
| *GRM1* | 6q24 | mGluR1 | Metabotropic glutamate receptor 5 |
| *GRM5* | 11q14.3 | mGluR5 | Metabotropic glutamate receptor 1 |
| *HOMER1* | 5q14.2 | Homer1 | PSD scaffolding protein |
| *HRAS* | 11p15.5 | H-Ras | small G protein –  activates Ras/ERK cascade |
| *MAP2K1* | 15q22.1-22.33 | MEK1 | Ras/ERK cascade kinase |
| *MAP2K2* | 19p13.3 | MEK2 | Ras/ERK cascade kinase |
| *PIK3CA* | 3q26.3 | PI3K | PI3K catalytic subunit–  activates PI3K/mTOR cascade |
| *PIK3R1* | 5q13.1 | PI3K | PI3K regulatory subunit |
| *PTEN* | 10q23.3 | PTEN | PIP_3_ phosphatase –  antagonizes PI3K |
| *RAF1* | 3p25 | Raf | Ras/ERK cascade kinase |
| *RHEB* | 7q36 | Rheb | small G protein -  activates mTOR |
| *SHANK3* | 22q13.3 | Shank3 | PSD scaffolding protein |
| *TSC1* | 9q34 | TSC1 | Rheb GAP –  represses mTOR |
| *TSC2* | 16p13.3 | TSC2 | Rheb GAP –  represses mTOR |
| *UBE3A* | 15q11.2 | Ube3a | E3 ubiquitin ligase |

Gene and protein names, cytogenetic localization and protein function are listed for the 18 genes encoding mGluR pathway components that were subjected to next-generation sequencing in a cohort of autism cases and controls in this study.
