# Supplementary material for: High-Throughput Sequencing of mGluR Signaling Pathway Genes Reveals Enrichment of Rare Variants in Autism
Source: PLoS One. 2012 Apr 27;7(4):e35003. doi: 10.1371/journal.pone.0035003 (PMC3338748; doi:10.1371/journal.pone.0035003)
Supplement: Table S2 — Cohort demographic and diagnostic characteristics. The demographic and diagnostic features of the AGRE and control cohorts analyzed in this study are summarized. (DOCX) [file pone.0035003.s003.docx]

|  | **AGRE** | **Control** |
| --- | --- | --- |
| Total number subjects | 290 | 300 |
| Males | 232 | 141 |
| Females | 58 | 159 |
| White, not Hispanic or Latino | 232 | 248 |
| Hispanic or Latino | 53 | 52 |
| Asian | 5 | 0 |
| Diagnosis of autism (ADI-R) | 290 | NA |
| Diagnosis of autism (ADOS) | 224 | NA |
| Diagnosis of ASD (ADOS) | 23 | NA |
| Affected sibling (including 20 twins) | 287 | NA |
| No affected sibling | 3 | NA |

The demographic and diagnostic features of the AGRE and control cohorts analyzed in this study are summarized.
