# Supplementary material for: High-Throughput Sequencing of mGluR Signaling Pathway Genes Reveals Enrichment of Rare Variants in Autism
Source: PLoS One. 2012 Apr 27;7(4):e35003. doi: 10.1371/journal.pone.0035003 (PMC3338748; doi:10.1371/journal.pone.0035003)
Supplement: Table S3 — Rare variants detected in AGRE and/or control groups. All SNVs in 18 mGluR pathway genes that were concordantly detected on both NGS platforms are listed and annotated in terms of their location and consequence, occurrence in AGRE and/or control groups, minor allele frequency (maf), and functional significance as outlined in Fig. 2B. (PDF) [file pone.0035003.s004.pdf]

Table S3. Rare variants detected in AGRE and/or control groups

Gene	RNA Isoform	Nucleotide Change	Amino Acid Change	Consequence or Location	maf >=1%, presumed benign	maf <1%, presumed benign	maf <1%, NOT presumed benign	maf <1%, potentially deleterious
ARC	NM_015193.3	c.382G>A	D128N	missense		Both		
ARC	NM_015193.3	c.489C>T	D163D	synonymous	Both			
ARC	NM_015193.3	c.587C>G	P196R	missense			**AGRE**	**AGRE**
ARC	NM_015193.3	c.598G>A	G200S	missense			*Controls*	*Controls*
ARC	NM_015193.3	c.605A>T	D202V	missense			**AGRE**	**AGRE**
ARC	NM_015193.3	c.607G>A	G203R	missense		Both		
ARC	NM_015193.3	c.642C>T	F214F	synonymous		Both		
ARC	NM_015193.3	c.747G>A	P249P	synonymous		*Controls*		
ARC	NM_015193.3	c.829G>A	G277S	missense			**AGRE**	**AGRE**
ARC	NM_015193.3	c.835C>T	G277S	synonymous		*Controls*		
ARC	NM_015193.3	c.858C>G	R286R	synonymous	Both			
ARC	NM_015193.3	c.870G>A	L290L	synonymous		*Controls*		
ARC	NM_015193.3	c.873G>A	P291P	synonymous		*Controls*		
ARC	NM_015193.3	c.885C>T	G295G	synonymous		*Controls*		
EIF4E	NM_001968.3	c.-21G>C		5'UTR		Both		
EIF4E	NM_001968.3	c.318A>G	K106K	synonymous		*Controls*		
EIF4E	NM_001968.3	c.349T>C	L117L	synonymous		*Controls*		
EIF4E	NM_001968.3	c.375C>T	D125D	synonymous	Both			
EIF4E	NM_001968.3	c.540-10T>G		intronic			**AGRE**	
EIF4E	NM_001968.3	c.622G>A	G208S	missense		Both		
FMR1	NM_002024.5	c.52-3C>T		intronic			*Controls*	
FMR1	NM_002024.5	c.271-19A>G		intronic		Both		
FMR1	NM_002024.5	c.413G>A	R138Q	missense			*Controls*	*Controls*
FMR1	NM_002024.5	c.414G>A	R138R	synonymous	Both			
FMR1	NM_002024.5	c.433G>T	A145S	missense	*Controls*			
FMR1	NM_002024.5	c.521A>G	N174S	missense			*Controls*	*Controls*
FMR1	NM_002024.5	c.990+14C>T		intronic	Both			
FMR1	NM_002024.5	c.1092T>C	H364H	synonymous		*Controls*		
FMR1	NM_002024.5	c.1737+8C>A		intronic		Both		
FMR1	NM_002024.5	c.1857C>T	D619D	synonymous		Both		
GRM1	NM_000838.3	c.98C>T	S33L	missense		Both		

GRM1	NM_000838.3	c.101C>G	S34C	missense		Both		
GRM1	NM_000838.3	c.624C>T	D208D	synonymous		**AGRE**		
GRM1	NM_000838.3	c.726C>T		synonymous		*Controls*		
GRM1	NM_000838.3	c.950+7T>C		intronic		Both		
GRM1	NM_000838.3	c.1338C>T	D446D	synonymous		*Controls*		
GRM1	NM_000838.3	c.1488C>T	H496H	synonymous		*Controls*		
GRM1	NM_000838.3	c.1602+9C>G		intronic			*Controls*	
GRM1	NM_000838.3	c.1643C>T	T548M	missense		Both		
GRM1	NM_000838.3	c.1750C>T	R584C	missense			*Controls*	*Controls*
GRM1	NM_000838.3	c.1882C>G	R628G	missense			**AGRE**	**AGRE**
GRM1	NM_000838.3	c.1983C>T	R661R	synonymous		**AGRE**		
GRM1	NM_000838.3	c.1993G>A	G665S	missense		Both		
GRM1	NM_000838.3	c.2026G>A	V676M	missense			*Controls*	*Controls*
GRM1	NM_000838.3	c.2051G>A	R684H	missense			**AGRE**	**AGRE**
GRM1	NM_000838.3	c.2185C>A	P729T	missense	Both			
GRM1	NM_000838.3	c.2253G>A		synonymous		*Controls*		
GRM1	NM_000838.3	c.2334C>T		synonymous		Both		
GRM1	NM_000838.3	c.2340C>T	N780N	synonymous		**AGRE**		
GRM1	NM_000838.3	c.2581G>A	G861S	missense		Both		
GRM1	NM_000838.3	c.2651G>A	G884E	missense	*Controls*			
GRM1	NM_000838.3	c.2725A>C	M909L	missense		Both		
GRM1	NM_000838.3	c.2785G>A	V929I	missense	**AGRE**			
GRM1	NM_000838.3	c.2793G>A	K931K	synonymous	Both			
GRM1	NM_000838.3	c.2859C>T	T953T	synonymous		**AGRE**		
GRM1	NM_000838.3	c.2922T>C	P974P	synonymous		Both		
GRM1	NM_000838.3	c.2977T>C	S993P	missense	Both			
GRM1	NM_000838.3	c.3016G>A	E1006K	missense		Both		
GRM1	NM_000838.3	c.3069C>T		synonymous		Both		
GRM1	NM_000838.3	c.3106G>A	G1036R	missense			*Controls*	*Controls*
GRM1	NM_000838.3	c.3107G>T	G1036V	missense			*Controls*	*Controls*
GRM1	NM_000838.3	c.3168T>G		synonymous	Both			
GRM1	NM_000838.3	c.3213T>G		synonymous	Both			
GRM1	NM_000838.3	c.3214C>G	P1072A	missense			*Controls*	*Controls*
GRM1	NM_000838.3	c.3219G>A	Q1073Q	synonymous		Both		
GRM1	NM_000838.3	c.3357G>C	T1119T	synonymous		Both		

GRM1	NM_000838.3	c.3495C>A	P1165P	synonymous	Both			
GRM1	NM_000838.3	c.3600C>T		3'UTR			*Controls*	*Controls*
GRM5	NM_000842.3	c.-8T>G		5'UTR	Both			
GRM5	NM_000842.3	c.-17T>G		5'UTR	Both			
GRM5	NM_000842.3	c.20T>A	L7Q	missense			*Controls*	*Controls*
GRM5	NM_000842.3	c.87T>C	A29A	synonymous		**AGRE**		
GRM5	NM_000842.3	c.95C>T	P32L	missense	Both			
GRM5	NM_000842.3	c.96G>A	P32P	synonymous		Both		
GRM5	NM_000842.3	c.151A>G	K51E	missense	Both			
GRM5	NM_000842.3	c.177G>A	A59A	synonymous	Both			
GRM5	NM_000842.3	c.189G>A		synonymous		*Controls*		
GRM5	NM_000842.3	c.291C>T	D97D	synonymous	Both			
GRM5	NM_000842.3	c.360A>G	S120S	synonymous	Both			
GRM5	NM_000842.3	c.386G>C	C129S	missense	*Controls*			
GRM5	NM_000842.3	c.410T>C	F137S	missense	Both			
GRM5	NM_000842.3	c.412C>T	R138C	missense		Both		
GRM5	NM_000842.3	c.450C>T	G150G	synonymous	Both			
GRM5	NM_000842.3	c.527G>T	S176I	missense	Both			
GRM5	NM_000842.3	c.601G>T	A201S	missense	Both			
GRM5	NM_000842.3	c.651G>A	V217V	synonymous	Both			
GRM5	NM_000842.3	c.657A>C		synonymous		Both		
GRM5	NM_000842.3	c.661+11T>C		intronic	Both			
GRM5	NM_000842.3	c.661+3A>G		intronic	Both			
GRM5	NM_000842.3	c.727G>T	A243S	missense			**AGRE**	**AGRE**
GRM5	NM_000842.3	c.738C>A	Y246*	nonsense		Both		
GRM5	NM_000842.3	c.827G>A	C276Y	missense	Both			
GRM5	NM_000842.3	c.846G>A	T282T	synonymous		**AGRE**		
GRM5	NM_000842.3	c.887C>T	A296V	missense	Both			
GRM5	NM_000842.3	c.911+10A>G		intronic	Both			
GRM5	NM_000842.3	c.911+3G>A		intronic			**AGRE**	
GRM5	NM_000842.3	c.1167A>G	T389T	synonymous		**AGRE**		
GRM5	NM_000842.3	c.1206C>T	N402N	synonymous		Both		
GRM5	NM_000842.3	c.1295T>C	I432T	missense			*Controls*	*Controls*
GRM5	NM_000842.3	c.1358C>T	T453M	missense		Both		
GRM5	NM_000842.3	c.1394+14A>G		intronic			*Controls*	

GRM5	NM_000842.3	c.1417G>C	E473Q	missense			**AGRE**	**AGRE**
GRM5	NM_000842.3	c.1496T>C	V499A	missense			*Controls*	*Controls*
GRM5	NM_000842.3	c.1563+8G>A		intronic			**AGRE**	
GRM5	NM_000842.3	c.1691-4G>T		intronic			**AGRE**	
GRM5	NM_000842.3	c.1731C>T		synonymous		*Controls*		
GRM5	NM_000842.3	c.2034G>A	K678K	synonymous		Both		
GRM5	NM_000842.3	c.2127T>A	V709V	synonymous		**AGRE**		
GRM5	NM_000842.3	c.2220T>G		synonymous		*Controls*		
GRM5	NM_000842.3	c.2339C>T	T780M	missense			*Controls*	*Controls*
GRM5	NM_000842.3	c.2379T>C	F793F	synonymous		**AGRE**		
GRM5	NM_000842.3	c.2497G>A	V833M	missense			*Controls*	*Controls*
GRM5	NM_000842.3	c.2630+10G>A		intronic			**AGRE**	
GRM5	NM_000842.3	c.2652G>A	T884T	synonymous		**AGRE**		
GRM5	NM_000842.3	c.2705C>T	S902F	missense		Both		
GRM5	NM_000842.3	c.2942C>A	P981H	missense		Both		
GRM5	NM_000842.3	c.2943C>A	P981P	synonymous		Both		
GRM5	NM_000842.3	c.2943C>T	P981P	synonymous	Both			
GRM5	NM_000842.3	c.2945C>A	A982E	missense		Both		
GRM5	NM_000842.3	c.2945C>T	A982V	missense	Both			
GRM5	NM_000842.3	c.2954G>A	R985H	missense		Both		
GRM5	NM_000842.3	c.2954G>T	R985L	missense	**AGRE**			
GRM5	NM_000842.3	c.2964G>A		synonymous		*Controls*		
GRM5	NM_000842.3	c.2968A>G	I990V	missense			*Controls*	*Controls*
GRM5	NM_000842.3	c.3123C>T	S1041S	synonymous		**AGRE**		
GRM5	NM_000842.3	c.3157C>T	P1053S	missense		Both		
GRM5	NM_000842.3	c.3279A>C		synonymous	Both			
GRM5	NM_000842.3	c.3349G>A	E1117K	missense		Both		
GRM5	NM_000842.3	c.3349G>T	E1117*	nonsense	**AGRE**			
GRM5	NM_000842.3	c.3351G>A	E1117E	synonymous		Both		
GRM5	NM_000842.3	c.3354T>A	A1118A	synonymous		Both		
GRM5	NM_000842.3	c.3503T>C	L1168P	missense			**AGRE**	**AGRE**
HOMER1	NM_004272.3	c.6-4G>T		intronic	Both			
HOMER1	NM_004272.3	c.45A>G	Q15Q	synonymous	Both			
HOMER1	NM_004272.3	c.162+19G>T		intronic			**AGRE**	
HOMER1	NM_004272.3	c.195G>T	M65I	missense			**AGRE**	**AGRE**

HOMER1	NM_004272.3	c.290C>T	S97L	missense			**AGRE**	**AGRE**
HOMER1	NM_004272.3	c.295-12C>T		intronic	Both			
HOMER1	NM_004272.3	c.387+18A>G		intronic	Both			
HOMER1	NM_004272.3	c.400G>A	G134R	missense		Both		
HOMER1	NM_004272.3	c.425C>T	P142L	missense			**AGRE**	**AGRE**
HOMER1	NM_004272.3	c.511T>C	L171L	synonymous		*Controls*		
HOMER1	NM_004272.3	c.528-11T>G		intronic			**AGRE**	
HOMER1	NM_004272.3	c.968G>A	R323H	missense			**AGRE**	**AGRE**
HOMER1	NM_004272.3	c.1080C>T		3'UTR			**AGRE**	**AGRE**
HRAS	NM_176795.3	c.-10C>T		5'UTR	Both			
HRAS	NM_176795.3	c.81T>C	H27H	synonymous	Both			
HRAS	NM_176795.3	c.111+15G>A		intronic	Both			
HRAS	NM_176795.3	c.249C>T		synonymous		Both		
HRAS	NM_176795.3	c.357C>T		synonymous		*Controls*		
HRAS	NM_176795.3	c.383G>A	R128Q	missense			**AGRE**	**AGRE**
HRAS	NM_176795.3	c.498C>G	P166P	synonymous		**AGRE**		
MAP2K1	NM_002755.3	c.-2A>G		5'UTR		Both		
MAP2K1	NM_002755.3	c.292-3C>T		intronic		Both		
MAP2K1	NM_002755.3	c.315C>T	P105P	synonymous		**AGRE**		
MAP2K1	NM_002755.3	c.371C>T	P124L	missense		Both		
MAP2K1	NM_002755.3	c.438+20C>T		intronic	Both			
MAP2K1	NM_002755.3	c.711G>A	G237G	synonymous	*Controls*	**AGRE**		
MAP2K1	NM_002755.3	c.1003C>G	Q335E	missense			*Controls*	*Controls*
MAP2K1	NM_002755.3	c.1023-8C>T		intronic	Both			
MAP2K2	NM_030662.3	c.90C>T		synonymous		*Controls*		
MAP2K2	NM_030662.3	c.93-20A>C		intronic			**AGRE**	
MAP2K2	NM_030662.3	c.100C>T	L34L	synonymous		*Controls*		
MAP2K2	NM_030662.3	c.192C>T	V64V	synonymous	Both			
MAP2K2	NM_030662.3	c.258C>A	V86V	synonymous		**AGRE**		
MAP2K2	NM_030662.3	c.303+18G>A		intronic	*Controls*			
MAP2K2	NM_030662.3	c.405G>C	G135G	synonymous		Both		
MAP2K2	NM_030662.3	c.420C>T	D140D	synonymous		**AGRE**		
MAP2K2	NM_030662.3	c.450+15G>T		intronic		Both		
MAP2K2	NM_030662.3	c.453C>T	D151D	synonymous	Both			
MAP2K2	NM_030662.3	c.454G>A	G152S	missense		Both		

MAP2K2	NM_030662.3	c.498C>T	P166P	synonymous		Both		
MAP2K2	NM_030662.3	c.528+20A>G		intronic		Both		
MAP2K2	NM_030662.3	c.528G>A	A176A	synonymous		**AGRE**		
MAP2K2	NM_030662.3	c.580+6G>A		intronic		Both		
MAP2K2	NM_030662.3	c.581-1G>T		splice-site			**AGRE**	**AGRE**
MAP2K2	NM_030662.3	c.660C>A	I220I	synonymous	Both			
MAP2K2	NM_030662.3	c.678C>T		synonymous		Both		
MAP2K2	NM_030662.3	c.690G>A	T230T	synonymous		*Controls*		
MAP2K2	NM_030662.3	c.846C>T	P282P	synonymous		Both		
MAP2K2	NM_030662.3	c.889C>T	R297W	missense			**AGRE**	**AGRE**
MAP2K2	NM_030662.3	c.903C>T	P301P	synonymous		Both		
MAP2K2	NM_030662.3	c.919+12A>G		intronic	Both			
MAP2K2	NM_030662.3	c.981C>T	N327N	synonymous		*Controls*		
MAP2K2	NM_030662.3	c.985-18C>T		intronic		Both		
MAP2K2	NM_030662.3	c.1074G>A		synonymous		Both		
MAP2K2	NM_030662.3	c.1140C>T	A380A	synonymous		*Controls*		
MAP2K2	NM_030662.3	c.1141G>C	G381R	missense			*Controls*	*Controls*
MAP2K2	NM_030662.3	c.1211C>T		3'UTR		Both		
MAP2K2	NM_030662.3	c.1217C>T		3'UTR			**AGRE**	**AGRE**
MAP2K2	NM_030662.3	c.1222C>T		3'UTR		Both		
MAP2K2	NM_030662.3	c.1227C>T		3'UTR	Both			
PIK3CA	NM_006218.2	c.50C>A	P17H	missense			*Controls*	*Controls*
PIK3CA	NM_006218.2	c.363C>T	I121I	synonymous	Both			
PIK3CA	NM_006218.2	c.466C>T	L156F	missense			*Controls*	*Controls*
PIK3CA	NM_006218.2	c.1060-17C>A		intronic	Both			
PIK3CA	NM_006218.2	c.1060-18C>A		intronic		Both		
PIK3CA	NM_006218.2	c.1143C>G	P381P	synonymous		**AGRE**		
PIK3CA	NM_006218.2	c.1173A>G	I391M	missense	Both			
PIK3CA	NM_006218.2	c.1544A>G	N515S	missense			**AGRE**	**AGRE**
PIK3CA	NM_006218.2	c.1747-13T>C		intronic	Both			
PIK3CA	NM_006218.2	c.1788A>G	E596E	synonymous		**AGRE**		
PIK3CA	NM_006218.2	c.1837G>A	G613S	missense	Both			
PIK3CA	NM_006218.2	c.1850G>A	R617Q	missense	Both			
PIK3CA	NM_006218.2	c.1876G>A	D626N	missense	Both			
PIK3CA	NM_006218.2	c.1911G>A	Q637Q	synonymous	Both			

PIK3CA	NM_006218.2	c.1912-12G>T		intronic	Both			
PIK3CA	NM_006218.2	c.1930T>C	Y644H	missense	Both			
PIK3CA	NM_006218.2	c.2016-12C>T		intronic	Both			
PIK3CA	NM_006218.2	c.2038G>C	V680L	missense	Both			
PIK3CA	NM_006218.2	c.2102A>C	H701P	missense		Both		
PIK3CA	NM_006218.2	c.2119G>A	E707K	missense	Both			
PIK3CA	NM_006218.2	c.2155C>G	L719V	missense	Both			
PIK3CA	NM_006218.2	c.2187+16T>C		intronic	Both			
PIK3CA	NM_006218.2	c.2187+1G>T		splice-site	Both			
PIK3CA	NM_006218.2	c.2294+19C>T		intronic			**AGRE**	
PIK3CA	NM_006218.2	c.2439A>G	T813T	synonymous		**AGRE**		
PIK3CA	NM_006218.2	c.3075C>T	T1025T	synonymous		**AGRE**		
PIK3R1	NM_181523.1	c.219C>T	Y73Y	synonymous	Both			
PIK3R1	NM_181523.1	c.334+14A>C		intronic	Both			
PIK3R1	NM_181523.1	c.427+5C>A		intronic			*Controls*	
PIK3R1	NM_181523.1	c.428-18G>A		intronic			*Controls*	
PIK3R1	NM_181523.1	c.621T>C	I207I	synonymous	Both			
PIK3R1	NM_181523.1	c.634+8T>C		intronic		Both		
PIK3R1	NM_181523.1	c.635-9A>G		intronic			**AGRE**	
PIK3R1	NM_181523.1	c.687G>A	S229S	synonymous		Both		
PIK3R1	NM_181523.1	c.837-13C>T		intronic			**AGRE**	
PIK3R1	NM_181523.1	c.889G>A	E297K	missense			**AGRE**	**AGRE**
PIK3R1	NM_181523.1	c.902G>A	R301Q	missense		Both		
PIK3R1	NM_181523.1	c.917-10T>G		intronic			**AGRE**	
PIK3R1	NM_181523.1	c.978G>A	M326I	missense	Both			
PIK3R1	NM_181523.1	c.1176C>T	F392F	synonymous	Both			
PIK3R1	NM_181523.1	c.1426-20T>G		intronic			*Controls*	
PIK3R1	NM_181523.1	c.2109T>C	L703L	synonymous		Both		
PIK3R1	NM_181523.1	c.2178C>T		3'UTR	Both			
PTEN	NM_000314.4	c.132C>T	G44G	synonymous		Both		
PTEN	NM_000314.4	c.425G>A	R142Q	missense		Both		
PTEN	NM_000314.4	c.802-17T>C		intronic		Both		
PTEN	NM_000314.4	c.802-18C>T		intronic	Both			
PTEN	NM_000314.4	c.802-19T>C		intronic		Both		
RAF1	NM_002880.3	c.119G>A	R40H	missense			*Controls*	*Controls*

RAF1	NM_002880.3	c.122G>A	R41Q	missense			**AGRE**	**AGRE**
RAF1	NM_002880.3	c.356C>T	A119V	missense			**AGRE**	**AGRE**
RAF1	NM_002880.3	c.923C>T	P308L	missense			*Controls*	*Controls*
RAF1	NM_002880.3	c.1422A>C		synonymous		Both		
RAF1	NM_002880.3	c.1668+19G>T		intronic		Both		
RAF1	NM_002880.3	c.1669-13T>C		intronic		Both		
RAF1	NM_002880.3	c.1755A>G	V585V	synonymous		Both		
RAF1	NM_002880.3	c.1830A>G	Q610Q	synonymous		Both		
RAF1	NM_002880.3	c.1941C>T	V647V	synonymous	**AGRE**	*Controls*		
RHEB	NM_005614.3	c.333-7C>T		intronic	Both			
RHEB	NM_005614.3	c.514G>A	G172R	missense		Both		
SHANK3	NM_001080420.1	c.64-18G>A		intronic		Both		
SHANK3	NM_001080420.1	c.448+16G>A		intronic	Both			
SHANK3	NM_001080420.1	c.522C>T		synonymous		**AGRE**		
SHANK3	NM_001080420.1	c.612C>A	D204E	missense			**AGRE**	**AGRE**
SHANK3	NM_001080420.1	c.734T>C	I245T	missense	Both			
SHANK3	NM_001080420.1	c.763C>T	H255Y	missense			**AGRE**	**AGRE**
SHANK3	NM_001080420.1	c.769-7C>G		intronic			**AGRE**	
SHANK3	NM_001080420.1	c.885+5G>T		intronic		Both		
SHANK3	NM_001080420.1	c.886-8C>T		intronic		Both		
SHANK3	NM_001080420.1	c.891C>T	S297S	synonymous		Both		
SHANK3	NM_001080420.1	c.898C>T	R300C	missense			**AGRE**	**AGRE**
SHANK3	NM_001080420.1	c.920C>G	A307G	missense			**AGRE**	**AGRE**
SHANK3	NM_001080420.1	c.976G>A	A326T	missense			*Controls*	*Controls*
SHANK3	NM_001080420.1	c.1030+13C>T		intronic			*Controls*	
SHANK3	NM_001080420.1	c.1031-14C>T		intronic			**AGRE**	
SHANK3	NM_001080420.1	c.1074G>A		synonymous		*Controls*		
SHANK3	NM_001080420.1	c.1191C>T		synonymous		Both		
SHANK3	NM_001080420.1	c.1211G>A	R404Q	missense		Both		
SHANK3	NM_001080420.1	c.1245C>T		synonymous		*Controls*		
SHANK3	NM_001080420.1	c.1254G>A	E418E	synonymous		**AGRE**		
SHANK3	NM_001080420.1	c.1293A>G		synonymous		*Controls*		
SHANK3	NM_001080420.1	c.1305-15G>A		intronic		Both		
SHANK3	NM_001080420.1	c.1305-16G>A		intronic		Both		
SHANK3	NM_001080420.1	c.1305-17G>A		intronic		Both		

SHANK3	NM_001080420.1	c.1315C>T	P439S	missense			**AGRE**	**AGRE**
SHANK3	NM_001080420.1	c.1332G>A		synonymous		Both		
SHANK3	NM_001080420.1	c.1334G>A	G445E	missense		Both		
SHANK3	NM_001080420.1	c.1335G>T		synonymous		**AGRE**		
SHANK3	NM_001080420.1	c.1336G>A	G446R	missense		Both		
SHANK3	NM_001080420.1	c.1337G>A	G446E	missense		Both		
SHANK3	NM_001080420.1	c.1337G>C	G446A	missense		Both		
SHANK3	NM_001080420.1	c.1337G>T	G446V	missense			**AGRE**	**AGRE**
SHANK3	NM_001080420.1	c.1340C>T	A447V	missense		Both		
SHANK3	NM_001080420.1	c.1660+18T>C		intronic	Both			
SHANK3	NM_001080420.1	c.1661-8C>T		intronic		Both		
SHANK3	NM_001080420.1	c.1717G>A	D573N	missense		Both		
SHANK3	NM_001080420.1	c.1736+18G>A		intronic	Both			
SHANK3	NM_001080420.1	c.1819+16T>A		intronic		Both		
SHANK3	NM_001080420.1	c.1891G>A	V631M	missense		Both		
SHANK3	NM_001080420.1	c.1911G>A		synonymous		Both		
SHANK3	NM_001080420.1	c.1944+19G>C		intronic	Both			
SHANK3	NM_001080420.1	c.1947G>T	V649V	synonymous		*Controls*		
SHANK3	NM_001080420.1	c.1968G>A	K656K	synonymous		*Controls*		
SHANK3	NM_001080420.1	c.2077+10C>A		intronic			**AGRE**	
SHANK3	NM_001080420.1	c.2079C>T		synonymous		Both		
SHANK3	NM_001080420.1	c.2084C>T	P695L	missense		Both		
SHANK3	NM_001080420.1	c.2091C>G	P697P	synonymous		Both		
SHANK3	NM_001080420.1	c.2101C>T	P701S	missense		Both		
SHANK3	NM_001080420.1	c.2102C>T	P701L	missense		Both		
SHANK3	NM_001080420.1	c.2119C>T		synonymous		*Controls*		
SHANK3	NM_001080420.1	c.2158+10G>A		intronic		Both		
SHANK3	NM_001080420.1	c.2183-20G>A		intronic		Both		
SHANK3	NM_001080420.1	c.2194G>A	E732K	missense		Both		
SHANK3	NM_001080420.1	c.2209G>A	A737T	missense	Both			
SHANK3	NM_001080420.1	c.2223G>A	T741T	synonymous		Both		
SHANK3	NM_001080420.1	c.2259C>T	A753A	synonymous		Both		
SHANK3	NM_001080420.1	c.2313+20G>A		intronic			**AGRE**	
SHANK3	NM_001080420.1	c.2358G>A	P786P	synonymous		Both		
SHANK3	NM_001080420.1	c.2359G>A	G787S	missense		Both		

SHANK3	NM_001080420.1	c.2398+11G>A		intronic			**AGRE**	
SHANK3	NM_001080420.1	c.249G>T	T83T	synonymous		**AGRE**		
SHANK3	NM_001080420.1	c.3360C>T		synonymous		**AGRE**		
SHANK3	NM_001080420.1	c.3382C>T		synonymous		Both		
SHANK3	NM_001080420.1	c.3411C>T	S1137S	synonymous		Both		
SHANK3	NM_001080420.1	c.3568C>T	R1190C	missense			*Controls*	*Controls*
SHANK3	NM_001080420.1	c.3585G>A	K1195K	synonymous	**AGRE**	*Controls*		
SHANK3	NM_001080420.1	c.3708G>A	E1236E	synonymous		**AGRE**		
SHANK3	NM_001080420.1	c.3761C>T	A1254V	missense			**AGRE**	**AGRE**
SHANK3	NM_001080420.1	c.3764C>T	P1255L	missense			**AGRE**	**AGRE**
SHANK3	NM_001080420.1	c.3765G>A		synonymous		**AGRE**		
SHANK3	NM_001080420.1	c.3836C>T	P1279L	missense			**AGRE**	**AGRE**
SHANK3	NM_001080420.1	c.3850C>T	P1284S	missense			*Controls*	*Controls*
SHANK3	NM_001080420.1	c.3927C>T	S1309S	synonymous		Both		
SHANK3	NM_001080420.1	c.4025C>T	P1342L	missense			**AGRE**	**AGRE**
SHANK3	NM_001080420.1	c.4050C>T		synonymous		**AGRE**		
SHANK3	NM_001080420.1	c.4082C>T	P1361L	missense		Both		
SHANK3	NM_001080420.1	c.4149C>T	D1383D	synonymous		Both		
SHANK3	NM_001080420.1	c.4325C>T	P1442L	missense		Both		
SHANK3	NM_001080420.1	c.4362G>A		synonymous		Both		
SHANK3	NM_001080420.1	c.4368C>T	S1456S	synonymous		**AGRE**		
SHANK3	NM_001080420.1	c.4405G>C	G1469R	missense			**AGRE**	**AGRE**
SHANK3	NM_001080420.1	c.4406G>A	G1469E	missense		Both		
SHANK3	NM_001080420.1	c.4406G>T	G1469V	missense			**AGRE**	**AGRE**
SHANK3	NM_001080420.1	c.4479C>T		synonymous		**AGRE**		
SHANK3	NM_001080420.1	c.4490G>A	R1497Q	missense			**AGRE**	**AGRE**
SHANK3	NM_001080420.1	c.4501G>A	G1501R	missense		Both		
SHANK3	NM_001080420.1	c.4641C>T	I1547I	synonymous		**AGRE**		
SHANK3	NM_001080420.1	c.4652+12G>A		intronic		Both		
SHANK3	NM_001080420.1	c.4669G>A	G1557S	missense		Both		
SHANK3	NM_001080420.1	c.4708G>A	G1570S	missense		Both		
SHANK3	NM_001080420.1	c.4720G>A	G1574R	missense			**AGRE**	**AGRE**
SHANK3	NM_001080420.1	c.4995C>T		synonymous		Both		
SHANK3	NM_001080420.1	c.5071G>A	D1691N	missense		Both		
SHANK3	NM_001080420.1	c.5089C>T	H1697Y	missense		Both		

SHANK3	NM_001080420.1	c.5098G>A	E1700K	missense		Both		
SHANK3	NM_001080420.1	c.5106C>T		synonymous		**AGRE**		
SHANK3	NM_001080420.1	c.5164G>A	D1722N	missense		Both		
TSC1	NM_000368.4	c.-7C>T		5'UTR		Both		
TSC1	NM_000368.4	c.201A>G	P67P	synonymous		**AGRE**		
TSC1	NM_000368.4	c.210+18A>G		intronic			**AGRE**	
TSC1	NM_000368.4	c.346T>G	L116V	missense			**AGRE**	**AGRE**
TSC1	NM_000368.4	c.692C>T	P231L	missense			**AGRE**	**AGRE**
TSC1	NM_000368.4	c.871G>A	D291N	missense		Both		
TSC1	NM_000368.4	c.965T>C	M322T	missense	Both			
TSC1	NM_000368.4	c.1006C>T	R336W	missense			**AGRE**	**AGRE**
TSC1	NM_000368.4	c.1178C>T	T393I	missense			**AGRE**	**AGRE**
TSC1	NM_000368.4	c.1335A>G	E445E	synonymous	Both			
TSC1	NM_000368.4	c.1342C>T	P448S	missense			**AGRE**	**AGRE**
TSC1	NM_000368.4	c.1580A>G	Q527R	missense			**AGRE**	**AGRE**
TSC1	NM_000368.4	c.1701G>A		synonymous		*Controls*		
TSC1	NM_000368.4	c.1760A>G	K587R	missense	*Controls*			
TSC1	NM_000368.4	c.1795G>A	G599R	missense		Both		
TSC1	NM_000368.4	c.1960C>G	Q654E	missense			**AGRE**	**AGRE**
TSC1	NM_000368.4	c.1977G>A	A659A	synonymous		Both		
TSC1	NM_000368.4	c.1997+9C>T		intronic			*Controls*	
TSC1	NM_000368.4	c.2194C>T	H732Y	missense		Both		
TSC1	NM_000368.4	c.2285A>G	N762S	missense		Both		
TSC1	NM_000368.4	c.2392-13T>C		intronic			**AGRE**	
TSC1	NM_000368.4	c.2495C>A	S832Y	missense		Both		
TSC1	NM_000368.4	c.2626-3C>T		intronic			**AGRE**	
TSC1	NM_000368.4	c.2646C>T	A882A	synonymous		Both		
TSC1	NM_000368.4	c.2718A>C	Q906H	missense			**AGRE**	**AGRE**
TSC1	NM_000368.4	c.2829C>T	A943A	synonymous	Both			
TSC1	NM_000368.4	c.2865C>T	T955T	synonymous		Both		
TSC1	NM_000368.4	c.3042C>T	H1014H	synonymous		**AGRE**		
TSC1	NM_000368.4	c.3059C>T	T1020I	missense		Both		
TSC1	NM_000368.4	c.3103G>A	G1035S	missense		Both		
TSC1	NM_000368.4	c.3324C>T	G1108G	synonymous	*Controls*	**AGRE**		
TSC2	NM_000548.3	c.58G>A	G20R	missense		Both		

TSC2	NM_000548.3	c.138+20C>G		intronic			*Controls*	
TSC2	NM_000548.3	c.255C>T	V85V	synonymous		Both		
TSC2	NM_000548.3	c.272C>T	P91L	missense		Both		
TSC2	NM_000548.3	c.273G>A	P91P	synonymous		Both		
TSC2	NM_000548.3	c.273G>T	P91P	synonymous	**AGRE**	*Controls*		
TSC2	NM_000548.3	c.275A>C	E92A	missense		Both		
TSC2	NM_000548.3	c.275A>T	E92V	missense	Both			
TSC2	NM_000548.3	c.286G>A	E96K	missense		Both		
TSC2	NM_000548.3	c.348G>A		synonymous		*Controls*		
TSC2	NM_000548.3	c.363C>T		synonymous		Both		
TSC2	NM_000548.3	c.433G>A	A145T	missense			**AGRE**	**AGRE**
TSC2	NM_000548.3	c.482-3C>T		intronic	Both			
TSC2	NM_000548.3	c.618C>T	C206C	synonymous		**AGRE**		
TSC2	NM_000548.3	c.648+13C>T		intronic		Both		
TSC2	NM_000548.3	c.648+17C>T		intronic		Both		
TSC2	NM_000548.3	c.669C>T		synonymous		*Controls*		
TSC2	NM_000548.3	c.672C>T		synonymous		Both		
TSC2	NM_000548.3	c.729C>G		synonymous		Both		
TSC2	NM_000548.3	c.912G>A	W304*	nonsense		Both		
TSC2	NM_000548.3	c.944C>T	S315L	missense		Both		
TSC2	NM_000548.3	c.976-14G>C		intronic			*Controls*	
TSC2	NM_000548.3	c.1070C>T	A357V	missense			*Controls*	*Controls*
TSC2	NM_000548.3	c.1100G>A	R367Q	missense	Both			
TSC2	NM_000548.3	c.1110G>A	Q370Q	synonymous	*Controls*	**AGRE**		
TSC2	NM_000548.3	c.1116C>T		synonymous		*Controls*		
TSC2	NM_000548.3	c.1143G>A	R381R	synonymous		**AGRE**		
TSC2	NM_000548.3	c.1276C>T	L426L	synonymous		*Controls*		
TSC2	NM_000548.3	c.1281C>A	I427I	synonymous		Both		
TSC2	NM_000548.3	c.1292C>T	A431V	missense			**AGRE**	**AGRE**
TSC2	NM_000548.3	c.1320C>A	G440G	synonymous		*Controls*		
TSC2	NM_000548.3	c.1365C>T	S455S	synonymous		*Controls*		
TSC2	NM_000548.3	c.1377C>T	G459G	synonymous		*Controls*		
TSC2	NM_000548.3	c.1378G>A	A460T	missense		Both		
TSC2	NM_000548.3	c.1443+15G>A		intronic		Both		
TSC2	NM_000548.3	c.1578C>T	S526S	synonymous	Both			

TSC2	NM_000548.3	c.1600-14C>T		intronic	Both			
TSC2	NM_000548.3	c.1606G>A	A536T	missense		Both		
TSC2	NM_000548.3	c.1747G>A	A583T	missense	**AGRE**			
TSC2	NM_000548.3	c.1819G>A	A607T	missense		Both		
TSC2	NM_000548.3	c.1839+6G>A		intronic			**AGRE**	
TSC2	NM_000548.3	c.1912G>A	V638M	missense			**AGRE**	**AGRE**
TSC2	NM_000548.3	c.2031C>T		synonymous		Both		
TSC2	NM_000548.3	c.2049C>T		synonymous		*Controls*		
TSC2	NM_000548.3	c.2073C>T		synonymous		Both		
TSC2	NM_000548.3	c.2155T>C	Y719H	missense			**AGRE**	**AGRE**
TSC2	NM_000548.3	c.2545+10C>T		intronic			*Controls*	
TSC2	NM_000548.3	c.2546-12C>T		intronic	Both			
TSC2	NM_000548.3	c.2580T>C	F860F	synonymous	Both			
TSC2	NM_000548.3	c.2621C>T	P874L	missense			**AGRE**	**AGRE**
TSC2	NM_000548.3	c.2640-13C>T		intronic			*Controls*	
TSC2	NM_000548.3	c.2653A>G	I885V	missense			*Controls*	*Controls*
TSC2	NM_000548.3	c.2743-3C>A		intronic			*Controls*	
TSC2	NM_000548.3	c.2785G>A	E929K	missense			*Controls*	*Controls*
TSC2	NM_000548.3	c.2838-4A>G		intronic			*Controls*	
TSC2	NM_000548.3	c.2917G>A	E973K	missense		Both		
TSC2	NM_000548.3	c.3126G>T	P1042P	synonymous		**AGRE**		
TSC2	NM_000548.3	c.3132-13C>T		intronic			*Controls*	
TSC2	NM_000548.3	c.3145G>A	E1049K	missense		Both		
TSC2	NM_000548.3	c.3252C>G	D1084E	missense			**AGRE**	**AGRE**
TSC2	NM_000548.3	c.3327G>A	P1109P	synonymous		*Controls*		
TSC2	NM_000548.3	c.3429C>T		synonymous		*Controls*		
TSC2	NM_000548.3	c.3610+6G>A		intronic			*Controls*	
TSC2	NM_000548.3	c.3770C>T	A1257V	missense		Both		
TSC2	NM_000548.3	c.3815-15G>A		intronic		Both		
TSC2	NM_000548.3	c.3815-20C>T		intronic			*Controls*	
TSC2	NM_000548.3	c.3827C>T	S1276F	missense			**AGRE**	**AGRE**
TSC2	NM_000548.3	c.3883+5C>T		intronic		Both		
TSC2	NM_000548.3	c.3889G>A	A1297T	missense		Both		
TSC2	NM_000548.3	c.3914C>T	P1305L	missense			**AGRE**	**AGRE**
TSC2	NM_000548.3	c.3915G>A		synonymous		*Controls*		

TSC2	NM_000548.3	c.3919G>A	E1307K	missense		Both		
TSC2	NM_000548.3	c.3974G>A	G1325D	missense			**AGRE**	**AGRE**
TSC2	NM_000548.3	c.3986G>A	R1329H	missense		Both		
TSC2	NM_000548.3	c.4006-8C>T		intronic		Both		
TSC2	NM_000548.3	c.4047G>A	A1349A	synonymous		*Controls*		
TSC2	NM_000548.3	c.4051G>A	E1351K	missense			**AGRE**	**AGRE**
TSC2	NM_000548.3	c.4269G>A	L1423L	synonymous		Both		
TSC2	NM_000548.3	c.4285G>T	A1429S	missense		Both		
TSC2	NM_000548.3	c.4316G>A	G1439D	missense			**AGRE**	**AGRE**
TSC2	NM_000548.3	c.4341C>T		synonymous		**AGRE**		
TSC2	NM_000548.3	c.4403C>T	P1468L	missense		Both		
TSC2	NM_000548.3	c.4460C>G	S1487C	missense			**AGRE**	**AGRE**
TSC2	NM_000548.3	c.4506G>A	L1502L	synonymous		*Controls*		
TSC2	NM_000548.3	c.4536C>T	D1512D	synonymous		Both		
TSC2	NM_000548.3	c.4735G>A	G1579S	missense			*Controls*	*Controls*
TSC2	NM_000548.3	c.4959C>T	S1653S	synonymous	**AGRE**	*Controls*		
TSC2	NM_000548.3	c.4983C>T	T1661T	synonymous		*Controls*		
TSC2	NM_000548.3	c.4990-7C>T		intronic		Both		
TSC2	NM_000548.3	c.5028G>A	L1676L	synonymous		**AGRE**		
TSC2	NM_000548.3	c.5048T>G	V1683G	missense		Both		
TSC2	NM_000548.3	c.5050T>G	S1684A	missense	Both			
TSC2	NM_000548.3	c.5068+6G>A		intronic			*Controls*	
TSC2	NM_000548.3	c.5069-8C>T		intronic			**AGRE**	
TSC2	NM_000548.3	c.5131G>A	V1711M	missense			*Controls*	*Controls*
TSC2	NM_000548.3	c.5161-10A>C		intronic	Both			
TSC2	NM_000548.3	c.5175G>A		synonymous		**AGRE**		
TSC2	NM_000548.3	c.5202T>C	D1734D	synonymous	Both			
TSC2	NM_000548.3	c.5321G>C	S1774T	missense		Both		
TSC2	NM_000548.3	c.5359G>A	G1787S	missense		Both		
TSC2	NM_000548.3	c.5383C>T	R1795C	missense		Both		
TSC2	NM_000548.3	c.5397G>C	S1799S	synonymous	Both			
TSC2	NM_000548.3	c.5429G>A		3'UTR			**AGRE**	**AGRE**
TSC2	NM_000548.3	c.5450G>A		3'UTR			**AGRE**	**AGRE**
UBE3A	NM_130839.1	c.333C>G	N111K	missense			**AGRE**	**AGRE**
UBE3A	NM_130839.1	c.373T>C		synonymous		Both		

UBE3A	NM_130839.1	c.477A>T		synonymous		Both		
UBE3A	NM_130839.1	c.592G>A	A198T	missense	**AGRE**			
UBE3A	NM_130839.1	c.618A>T	A206A	synonymous		Both		
UBE3A	NM_130839.1	c.1269C>T		synonymous		**AGRE**		
UBE3A	NM_130839.1	c.1773A>G		synonymous		Both		
UBE3A	NM_130839.1	c.2124+9T>C		intronic			*Controls*	
UBE3A	NM_130839.1	c.2498+19G>C		intronic			*Controls*	
UBE3A	NM_130839.1	c.2618A>G		synonymous		Both		
UBE3A	NM_130839.1	c.2637T>C		3'UTR	Both			

All variants in 18 mGluR pathway genes that were concordantly detected on both NGS platforms are listed and annotated in terms of their location and consequence, occurrence in AGRE and/or control groups, minor allele frequency (maf), and functional significance as outlined in Fig. 2B.
